# Failures of the Fontan System in Univentricular Hearts and Mortality Risk in Heart Transplantation: A Systematic Review and Meta-Analysis

**DOI:** 10.3390/life11121363

**Published:** 2021-12-08

**Authors:** Horacio Márquez-González, Jose Gustavo Hernández-Vásquez, Montserrat Del Valle-Lom, Lucelli Yáñez-Gutiérrez, Miguel Klünder-Klünder, Eduardo Almeida-Gutiérrez, Solange Gabriela Koretzky

**Affiliations:** 1 Department of Clinical Research, Federico Gómez Children’s Hospital, Mexico City 06720, Mexico; hmarquez@himfg.edu.mx (H.M.-G.); gustavo.himfg@gmail.com (J.G.H.-V.); montsehimfg@gmail.com (M.D.V.-L.); mklunder@himfg.edu.mx (M.K.-K.); 2 Centro Médico Nacional Siglo XXI, IMSS, Department Congenital Heart Diseases, Mexico City 06720, Mexico; lucelli.yanezg@imss.gob.mx (L.Y.-G.); eduardo.almeida@imss.gob.mx (E.A.-G.); 3 Department of Clinical Research, Nacional de Cardiología “Ignacio Chávez”, Mexico City 14080, Mexico

**Keywords:** Fontan procedure, univentricular heart, heart transplantation, risk, mortality

## Abstract

The Fontan procedure (FP) is the standard surgical treatment for Univentricular heart diseases. Over time, the Fontan system fails, leading to pathologies such as protein-losing enteropathy (PLE), plastic bronchitis (PB), and heart failure (HF). FP should be considered as a transitional step to the final treatment: heart transplantation (HT). This systematic review and meta-analysis aims to establish the risk of death following HT according to the presence of FP complications. There was a total of 691 transplanted patients in the 18 articles, immediate survival 88% (*n* = 448), survival from 1 to 5 years of 78% (*n* = 427) and survival from 5.1 to 10 years of 69% (*n* = 208), >10 years 61% (*n* = 109). The relative risk (RR) was 1.12 for PLE (95% confidence interval [CI] = 0.89–1.40, *p* = 0.34), 1.03 for HF (0.7–1.51, *p* = 0.88), 0.70 for Arrhythmias (0.39–1.24, *p* = 0.22), 0.46 for PB (0.08–2.72, *p* = 0.39), and 5.81 for CKD (1.70–19.88, *p* = 0.005). In patients with two or more failures, the RR was 1.94 (0.99–3.81, *p* = 0.05). After FP, the risk of death after HT is associated with CKD and with the presence of two or more failures.

## 1. Introduction

Congenital heart disease (CHD) has an incidence of 8–10 cases per 1000 live births, and its overall survival is over 80% at 45 years [1,2], depending on the complexity of the malformations. In particular, univentricular heart diseases have the most complex spectrum of complications which occur at a high rate and are associated with lower survival.

The Fontan procedure (FP), or total cavopulmonary bypass, is the standard surgical treatment for univentricular heart diseases. It results in the creation of the Fontan System (FS), a circulatory rearrangement characterized by direct passive drainage of the systemic veins to the pulmonary circulation without the support of the subpulmonary ventricle. The cardiac mass is connected in series, dedicating its function as a pump exclusively to the systemic circulation. This is a palliative procedure, avoiding ventricular dysfunction by reducing the overload volume and controlling cyanosis [3,4].

The FP creates a new circuit by joining the systemic venous return and the pulmonary system, this is associate with a sudden lack of active right-heart pumping of blood into the pulmonary arterial tree, resulting in significant venous congestion, reduced ventricular filling, low cardiac output this conditions a pressure overload and a gradual remodeling in the venous vascular and lymphatic system [5], causing plastic bronchitis (PB) [6] and protein-losing enteropathy (PLE) [7,8]. The most frequent complication is ventricular dysfunction, which leads to death from heart failure (HF). Arrhythmias also develop, conditioned by the malformations of the conduction system and the flow redirection into the cavities [9]. The modified history of end-stage FP heart disease is accompanied by cardiac cirrhosis and cardio-renal syndrome [10]. Recently, there are authors who have investigated solutions to PF failures with venous Fontan ventricular assist such as Pekkan et al. [5]. Nevertheless, the FP is still considered as a transitional step to the final treatment, which is heart transplantation (HT) [11,12].

The proper functioning of the FP is the sum of morphological and hemodynamic variables; although they effectively increase survival, its primary objective is palliative. As time passes, the FP generates organic failures that deteriorate the quality of life, which can decrease the probability of success at the time of HT [13,14].

Analyzing the available scientific evidence is crucial in determining the mortality risk in transplant patients with univentricular physiology [15]. To this end, Tabarsi et al. published a meta-analysis [16] that reported a survival greater than 80% in the first year after HT in patients with FS. However, this study did not separately analyze survival according to the type of failure. Our study therefore aims to establish the risk of death after HT according to the presence of FS failures (PLE, PB, arrhythmias, HF, and chronic kidney disease [CKD]) in patients with univentricular heart disease.

## 2. Materials and Methods

A systematic review and meta-analysis of prognosis was carried based on the PRISMA 2020 statements [17]. 

### 2.1. Search Strategy and Data Sources

A systematic review of the literature was carried out with no starting date until 1 April 2021. The sources of information used were PUBMED, TRIP Database, International Clinical Trials Registry Platform (WHO), The Cochrane Library, Wiley, LILACS, and Google Scholar. In addition, aim-related systematic reviews were searched (snowball method) [16,18,19]. The reference lists of retrieved full-text articles were also searched to identify additional relevant studies. The search terms used were keywords or MESH terms (Supplementary Material Figure S1).

### 2.2. Eligibility Criteria

The articles and abstracts that fulfilled the PI(C)O criteria were included for further analysis. Population: Patients with HT due to CHD who had undergone FP or its variants; Intervention: Failure of FS; Comparator: PB, PLE, Arrhythmias, HF, CKD, and 2 or more failures; Outcome: death. Retrospective and prospective cohorts were acceptable study designs. Care was taken to select the articles that fulfilled the standards set in the Strengthening the Reporting of Observational Studies in Epidemiology (STROBE) statement [20].

Observational articles (longitudinal, cases and controls, and cohort) of patients with FP and HT, reporting the presence of at least one failure, were included. Publications made by the same author in the same hospital center were included, as long as they did not correspond to the same evaluation period. The exclusion criteria were: history of failure before FP or its takedown, multiorgan transplantation, and coexistence of autoimmune neoplastic diseases or other causes that were directly related to the outcome. Patients with cardiac retransplantation were also excluded. In the event that the articles met the selection criteria, but the data were insufficient to complete the analysis, the corresponding authors were contacted by email to request clarification of said data [21,22,23,24,25,26,27,28,29,30,31,32,33,34,35,36,37,38,39,40,41,42,43,44,45,46,47,48,49,50,51,52,53,54,55] in case that they sent information; it was then included in the review. 

Seven investigators (M.H., H.G., D.V.M., Y.L., K.M., A.E., and K.S.) independently reviewed all references identified through the literature search using a predefined protocol. Articles that did not meet inclusion criteria during the title and abstract analysis were excluded. The remaining articles were selected for full text review. When limited information was available from the abstract, the full text was always obtained. Included articles underwent a quality assessment by all the investigators.

Disagreements regarding the selection and quality assessment of articles were resolved through group discussion, and full consensus was achieved at each stage of review.

### 2.3. Data Extraction

Four investigators (M.H., K.S., H.G., and D.V.M.) independently extracted data from selected studies using a standardized electronic form in Excel. The following information was collected: author, year of publication, country, study design, total number of HT, total number of HT with fontan, deaths, and number of patients with each failure. 

### 2.4. Variables

Some variable of interest was death after HT. The exposure variables PLE, PB, arrythmias, and CKD were identified as the authors refer to the presence of the failure in the articles, authors definition of HF by cardiac catheterization in four articles [56,57,58,59], and by Echocardiogram in one [60], the rest of the articles was identified with the presence of HF by the authors. When the authors report the patient’s characteristics in a table format and it was possible to identify the presence of two or more failures it was included for the two or more failures analysis. 

### 2.5. Bias Control

Bias was evaluated with the GRADE tool (Grading of Recommendations, Assessment, Development, and Evaluation) [61] for observational studies, considering the following biases: adjusted confounders, validity of confounder measurement, analysis control, selection of participants, exposure assessment methods, exposure measurement, change in exposure status, outcome measurement, missing data, and missing exposure data. These were evaluated for each type of failure.

### 2.6. Statistical Analysis

A quantitative synthesis meta-analysis was performed using the Cochrane RevMan version 5.3 software for reviews, processing the data with a random effect method [62]. All outcomes that had at least 2 studies available for meta-analysis were finally reported. Risk of bias was assessed for each study proceeded as mandated by Cochrane standards. The relative risk (RR), confidence intervals (95% CI), and statistical significance were calculated using the Cochrane *X*^2^ test for each exposure variables. The study heterogeneity was assessed with the Tau test (due to small groups) and the *I*^2^ test. A value of *p* < 0.05 was considered statistically significant.

## 3. Results

**Figure 1:**
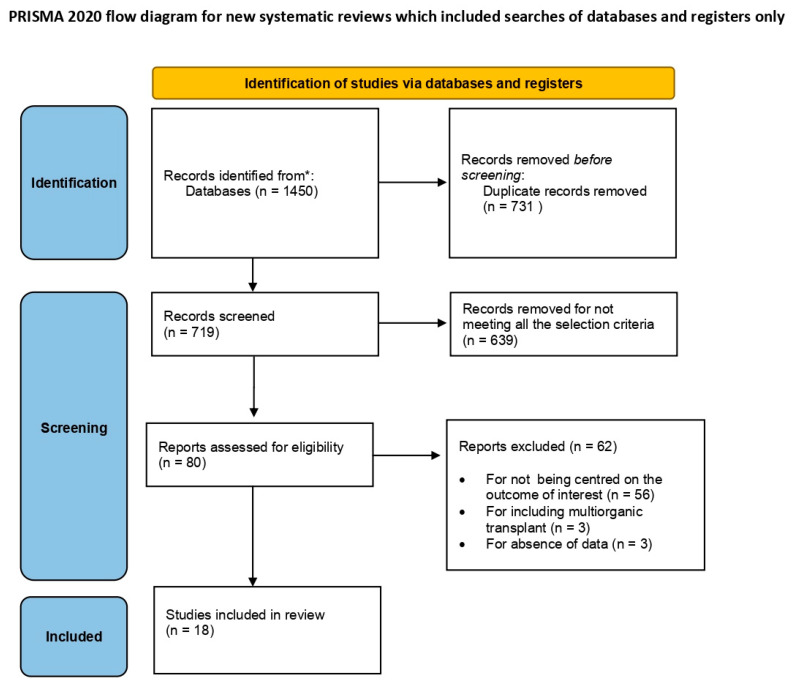
PRISMA flow: Selection of included studies.

From the keyword and Mesh Terms search, a total of 1450 abstracts were identified, of which 731 were removed as duplicates. The full texts of 80 articles were reviewed for their eligibility [6,7,9,10,11,12,13,14,15,16,18,19,21,22,23,24,25,26,27,28,29,30,31,32,33,34,35,36,37,38,39,40,41,42,43,44,56,57,58,59,60,63,64,65,66,67,68,69,70,71,72,73,74,75,76,77,78,79,80,81,82,83,84,85,86,87,88,89,90,91,92,93,94,95,96,97,98,99,100,101], and 18 were included in the final analysis [6,7,9,11,12,15,56,57,58,59,60,63,68,69,74,77,83,96] (Figure 1) [17].

**Figure 2:**
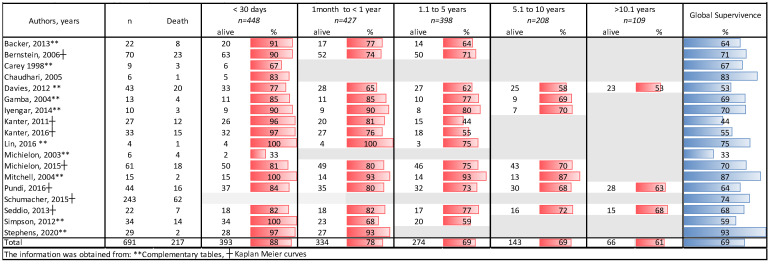
Analysis of survival by years in patients with the Fontan procedure and heart transplantation.

There was a total of 691 transplanted patients in the 18 articles (Table 1), with an immediate survival of 88% (*n* = 448), survival from 1 to 5 years of 78% (*n* = 427), survival from 5.1 to 10 years of 69% (*n* = 208), and survival > 10 years 61% (*n* = 109) (Figure 2).

**Figure 3:**
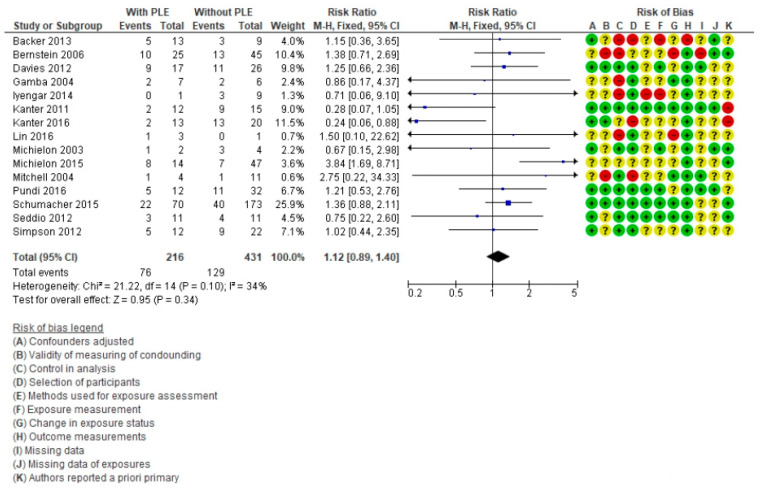
Forest plot comparing Fontan patients with protein-losing enteropathy (PLE) vs. without PLE for mortality post-cardiac transplant.

For the effect of PLE (Figure 3), 15 articles were included [6,7,9,11,12,15,57,58,59,60,68,74,77,83,96], with a total population of 647 patients (216 with PLE) of whom 205 died (76 with PLE). The RR was 1.12 (95% CI = 0.89–1.40, *p* = 0.34). The heterogeneity test showed a value of *I*^2^ = 34%. Authors Kanter [59,77] and Michielon [15,83] published studies from the same center which were included as there was an interval greater than five years between studies. This translates that the presence of PLE is not a risk factor for death in these patients.

**Figure 4:**
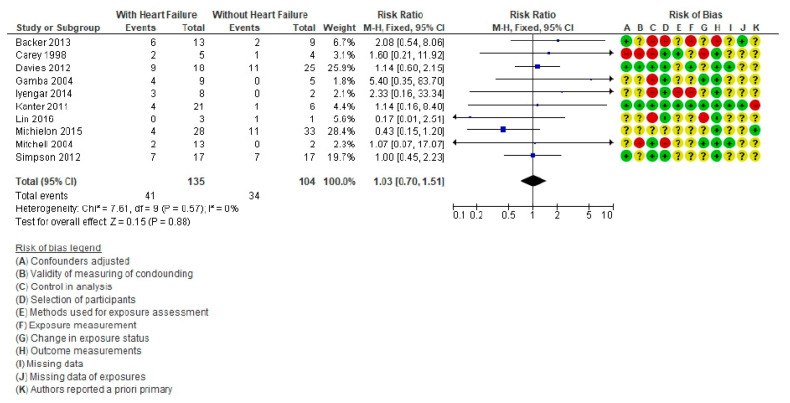
Forest plot comparing Fontan patients with cardiac failure vs. without cardiac failure for mortality post-cardiac transplant.

Regarding HF (Figure 4), 10 articles were included [6,9,12,15,57,58,60,69,74,77], with a total population of 239 patients (135 with HF) of whom 75 died (41 with HF). The RR was 1.03 (95% CI = 0.70–1.51, *p* = 0.88. The heterogeneity test showed a value of *I*^2^ = 0%. The presence of HF is not a risk factor for death in these patients.

**Figure 5:**
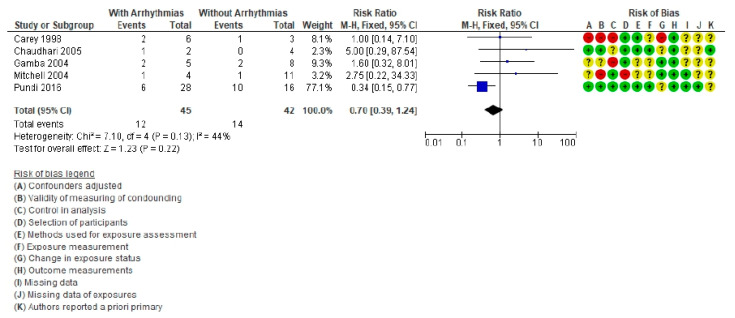
Forest plot comparing Fontan patients with arrhythmias vs. without arrhythmias for mortality post-cardiac transplant.

Five publications including arrhythmias were analyzed (Figure 5) [11,56,57,58,69], with a total of 87 patients (45 with arrhythmia) of whom 26 died (12 with arrhythmia). The RR was 0.70 (95% CI = 0.39–1.24, *p* = 0.13). Heterogeneity presented a value of *I*^2^ = 44%. The presences of arrhythmias are not a risk factor for death in these patients.

**Figure 6:**
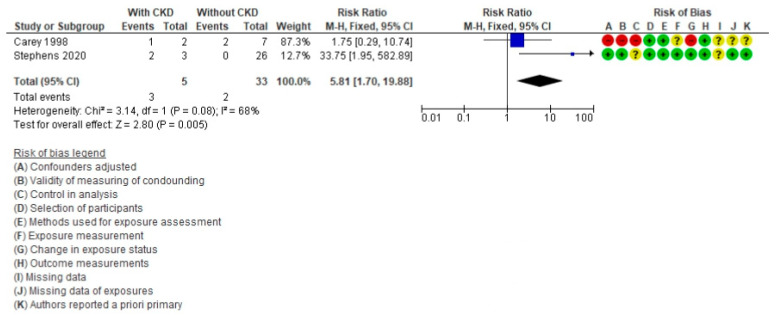
Forest plot comparing Fontan patients with chronic kidney disease (CKD) vs. without CKD for mortality post-cardiac transplant.

For the effect of CKD (Figure 6), two articles [63,69] were included, with a population of 38 patients (five with CKD) and a total of five deaths (three with CKD). The RR was 5.81 (95% CI = 1.70–19.88, *p* = 0.005). The heterogeneity test had an *I*^2^ value of 68%. The presence of CKD is a risk factor for death in patients with HT after FP.

**Figure 7:**
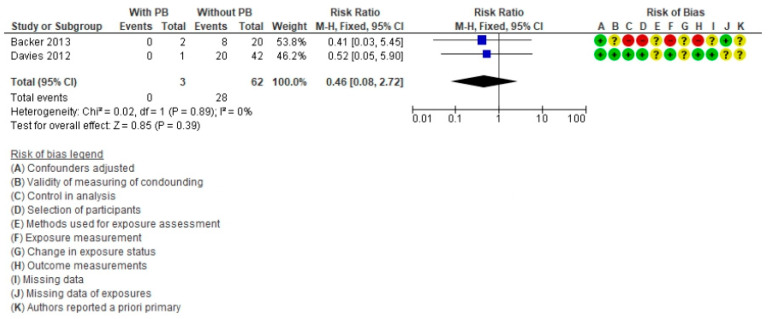
Forest plot comparing Fontan patients with plastic bronchitis (PB) vs. without PB for mortality post-cardiac transplant.

Two publications [6,12] were analyzed for PB, including 65 patients (three with PB) and no deaths (Figure 7). The estimated RR was 0.46 (95% CI = 0.08–2.72, *p* = 0.39), and the *I*^2^ heterogeneity was 0%. The presence of PB is not a risk factor for death in these patients.

**Figure 8:**
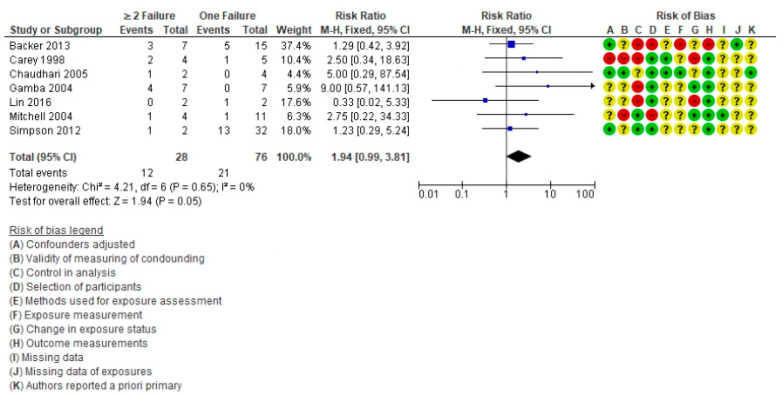
Forest plot comparing Fontan patients with a single failure vs. ≥2 failures for mortality post-cardiac transplant.

A subanalysis was carried out (Figure 8) including seven articles [6,9,56,57,58,60,69] with additional information on the coexistence of the failures. The total population was 104 subjects (28 with two or more failures) and there were 33 deaths, including 12 patients with two or more failures. The RR was 1.94 (95% CI = 0.99–3.81, *p* = 0.05) and the *I*^2^ heterogeneity was 0%. The coexistence of the failures are a risk factor for death in these patients.

## 4. Discussion

This systematic review and meta-analysis focuses on the effect of each failure of the FS as risk factors for mortality at the time of HT in different periods. It is the first meta-analysis that evaluates the individual risk of each failure for HT, the results are important since the findings showed that there is no association of death for failures of PLE, PB, HF, and arrhythmias, while the presence of Renal failure and the set of two failures if they represent risk and lower survival for patients who underwent HT after Fontan. In the 18 articles reviewed, these patients had a first-year survival of 79%, consistent with the findings of a 2017 meta-analysis by Tabarsi et al. which analyzed survival after HT in the presence of failures of the FS and found 80% survival at one year [16].

In the updated International Society for Heart and Lung Transplantation (ISHLT) database (as of 2020), there were a total of 30,130 patients with HT between 2004 and 2014. Of these, 1839 were related to CHD (one-year survival 78.3%), 16,444 to dilated cardiomyopathy (one-year survival 86.2%) and 12,247 to ischemic heart disease (survival of 84.3%) [102]. In this regard, patients with FP demonstrated 1.7-times higher risk of death from complications in the immediate postoperative period. The overall survival of HT performed from 5 to 10 years of age is greater than 60%, with a decrease after the first decade secondary to HF, which implies the need for retransplantation [103,104]. In the case of patients with FP, transplantation is useful to reverse the effects of failures, especially PLE and PB [13].

PLE is the consequence of increased pressure at different sites, such as the liver and splanchnic beds and the mesenteric network, which favors protein leakage into the intestinal lumen. Generally, patients who present with PLE also have other complications such as malnutrition, recurrent infections, and capillary leakage into the interstitial space. The present meta-analysis did not demonstrate a difference in mortality in the group of HT patients with PLE, which may be due to the fact that it is reversible when the etiological mechanism is removed.

PB occurs after FP with a frequency of 1–4%. Its etiological mechanism is similar to that of PLE, as well as lymphatic flow also being increased [105]. In this meta-analysis, the presence of PB was not associated with higher mortality. In the two articles analyzed, this complication was not reversible with HT as the lymphatic circulation does not fully improve; it is also associated with greater complications during immediate postoperative ventilatory support and pulmonary pressures are at high levels according to transplant criteria.

Of the patients included in this meta-analysis, 55.7% were classified with some of the criteria for HF. No differences in mortality were demonstrated in these patients at the time of transplantation. While the morphology of the systemic ventricle is associated with immediate mortality at the time of cavopulmonary bypass, no differences in the presentation of HF or other failures have been demonstrated when comparing a right or left morphology of the single ventricle [106,107]. Variables such as hospitalizations due to HF or sudden death events are directly associated with mortality in transplant patients [108], and these variables were not controlled in the meta-analysis.

Rhythm disorders are a frequent complication of FP. The most frequent are supraventricular tachyarrhythmias (approximately 60%) which include atrial fibrillation (40%), atrial flutter (17.2%) and atrial ectopic tachycardia (17.2%) [109]. The second most frequent group are second and third degree blocks; these are mostly treated with epicardial pacemakers, and can be accompanied by ventricular failure secondary to desynchrony [110]. In this work there was no association between death and the presence of arrhythmia, probably because the mechanism is completely resolved once the transplant has been performed, but complicated by the fact that in this analysis all rhythm disorders were grouped together [111].

The only failure associated with a statistically significant risk of death was CKD (RR = 5.8), which is conditioned by a mixed mechanism: first the pre-renal origin associated with HF and the distribution of fluid to the interstitial space and second by the intrarenal component due to prolonged diuretic intake [10,112]. Unlike PLE and PB [68,96], this lesion is irreversible and can become more acute in the immediate postoperative period, during ischemia and the postsurgical low-output syndrome.

Hollander et al. reported that after HT in patients with univentricular hearts with CKD, the 8% progressed to the renal stage [113], and during follow-up, 10% were expected to die in the next 5 years [114]. In the sub-analysis of this work, the two included articles did not specify the diagnostic method and stage of CKD, which may represent a misclassification bias. In this study, the analyses are based on comparing the failures with each other, which is likely the reason for the lack of significance in mortality differences. However, when focusing the analysis on the coexistence of two simultaneous failures, a value of RR = 1.94 was found, mostly explained by the coexistence of PLE and HF. It is important to consider the potential biases caused by the temporality of this phenomenon and the reference bias. It should also be noted that articles reporting multiorgan transplantation were excluded from the analysis.

There are some systemic diseases in which it is not yet clear whether they will benefit from a heart transplant such as: Kearns–Sayre syndrome that belongs to a group of neuromuscular disorders known as mitochondrial encephalomyopathies that typically involves the central nervous system, eyes, skeletal muscles, and heart [115]. Acute onset of congestive HF possible expression of a rare form of dilated cardiomyopathy; Fabry disease that is an X-linked lysosomal storage disorder caused by mutations in the a-galactosidase A gene (GLA) that leads to reduced or undetectable a-galactosidase A (AGAL) enzyme levels and progressive accumulation of glycolipids—primarily globotriaosylceramide (Gb3) and its deacylated form, lysoGb3, in cells throughout the body including vascular endothelial and smooth muscle cells and cardiomyocytes. Heart disease is present in all forms of Fabry disease, with different grades of organ involvement, and the concentric left ventricular (LV) hypertrophy [116]. Systemic sclerosis that is a systemic autoimmune disease of heterogeneous pathogenesis in which vascular, cutaneous, and internal organ fibrosis are prominent [117], in the literature there are reports of successful HT [115,116,117]; however, each case should never fail to be evaluated individually, for patients with PF and specifically for patients with Failing Fontan, there is no doubt that the treatment is HT and the fact of knowing that the presence of a single fault that could be PLE, PB, HF, and arrhythmias are not associated with a greater risk of death. It is new information in the literature since failures have never been evaluated in this way and it is very useful for patient care.

In patients with congenital heart disease, survival of HT is lower compared to other cardiomyopathies, much due to the fact that the clinical state is compromised, this occurs more frequently in patients with PF and its failures. In this meta-analysis we could identify that mortality is very similar to that reported by ISHLT. Most of the failures are not associated with mortality, except for CKD, which is not reversible. At the same time, we also found that the association of the presence of two or more failures has significant risk. These results are a first approach to having more information for the discrimination of patients who present greater risk and the selection of patients than if would benefit after the HT.

In this work, the random effect analysis was carried out, unlike the meta-analysis published by Tabarsi et al. [16], finding greater heterogeneity in the analysis of arrhythmias (*I*^2^ = 44), probably conditioned by the clinical diversity of the spectrum of electrical disorders considered as “ rhythm disturbances ” and CKD (*I*^2^ = 68%) explained by the methodological variability in its definition in the studies that included it.

We meta-analyzed the effect of five different failures in the risk of mortality in FP transplant patients, it is important to mention that some of the sub-analyzes were carried out with a limited number of articles and patients, for example, CKD with just three events and BP without deaths, these results demonstrate the need to carry out research studies focused on the standardization and follow-up of these failures. Also, misclassification bias is present in most of the included studies, for example for the PB the diagnosis information was obtained from the reference in the article by the authors not from medical records or from confirmatory tests such as alpha-1 antitrypsin in stool. Another example: an MRI or bronchoscopy study is required for the diagnosis of PB, which are not declared in the included studies, so there may be diagnostic confirmation bias. Another risk of bias in the included articles was the incomplete follow-up of the cohorts limiting the analysis to the first year after the HT, and in the case of publications by the same author who analyzed different periods it was not possible to identify whether there was patient repetition.

Other limitation of the study was that there are determining variables that intervene in the development of the disease, such as age at the time of the FP, pulmonary pressure, surgical technique, the type of treatment used, among others, that have not been weighted in this work because the variables measured in the included items are different from each other.

Another weakness to answer the objective is that the failure that is evaluated is compared with the group with the other failures; however, it cannot be performed in another way since if the patient does not present failure, there would be no indication for heart transplantation.

## 5. Conclusions

In conclusion, heart transplant in patients with a failing FP showed an immediate survival of 88%. PB, PLE, HF, and arrhythmias were not found to be associated with a greater risk of death, but the sum of two or more failures had an RR of 1.94 (95% CI = 0.99–3.81). The presence of CKD had an RR value of 5.81 (95% CI = 1.70–19.88). To fully understand the contribution of failures of FS to mortality, further studies with greater follow-up and clarity around the detection of failures is required.

## Figures and Tables

**Table 1:** Summary of studies included in the meta-analysis.

Author, Year	Country	Design	Overall Results			Outcomes				
			Transplantation	Fontan Population	Death	PLE	Arrythmia	HF	PB	CKD
Backer, 2013 [6]	USA	A retrospective study	206	22	8	5/13 (18%)	N/A	6/13 (46%)	0/2 (0%)	N/A
Bernstein, 2006 [68]	USA	A retrospective, multi-institutional study	1746	70	23	10/25 (40%)	N/A	N/A	N/A	N/A
Carey, 1998 [69]	UK	A retrospective review study in a single center, case series	46	9	3	N/A	2/6 (33%)	2/5 (40%)	N/A	1/2 (50%)
Chaudhar, 2005 [56]	UK	A retrospective review, a case series.	110	6	1	N/A	1/2 (50%)	N/A	N/A	N/A
Davies, 2012 [12]	USA	A retrospective review study	172	43	20	9/17 (53%)	N/A	9/18 (50%)	0/1 (0%)	N/A
Gamba, 2004 [57]	Italy	A retrospective review	575	13	4	2/7 (29%)	2/5 (40%)	4/9 (44%)	N/A	N/A
Iyengar, 2014 [74]	Australia	A retrospective study in a single center	111	10	3	0/1 (0%)	N/A	3/8 (38%)	NA	NA
Kanter, 2011 [77]	USA	A retrospective study in a Single center.	222	27	12	2/12 (17%)	N/A	4/21 (19%)	N/A	N/A
Kanter, 2016 [59]	USA	A retrospective study in a Single center.	311	33	15	2/13 (15%)	N/A	N/A	N/A	N/A
Lin, 2016 [60]	Taiwan	A retrospective study in a Single center, cases series.	513	4	1	1/3 (33%)	N/A	0/3 (0%)	N/A	N/A
Michielon, 2003 [83]	Italy	A Cohort study	25	6	8	1/2 (50%)	N/A	N/A	N/A	N/A
Michielon, 2015 [15]	Netherlands	A retrospective multicenter review.	61	61	18	11/14 (79%)	N/A	14/28 (50%)	N/A	N/A
Mitchell, 2004 [58]	USA	A retrospective study in a single center.	15	15	1	1/4 (25%)	1/4 (25%)	2/13 (15%)	N/A	N/A
Pundi, 2016 [11]	USA	A retrospective review, cohort study in a single center.	44	44	16	5/12 (42%)	6/28 (21%)	N/A	N/A	N/A
Schumacher, 2015 [7]	USA	A retrospective cohort study	3686	356	62	22/70 (31%)	N/A	N/A	N/A	N/A
Seddio, 2013 [96]	Italy	A retrospective cohort study	839	22	15	6/11 (55%)	N/A	N/A	N/A	N/A
Simpson, 2012 [9]	USA	A retrospective review study	34	34	11	5/12 (42%)	N/A	7/17 (41%)	N/A	N/A
Stephens, 2020 [63]	USA	A retrospective cohort study	153	32	5	N/A	N/A	N/A	N/A	2/3 (67%)

N/A: Not applicable.

## Data Availability

The datasets for this study are available by contacting the corresponding author.

## Supplementary Materials

The following are available online at https://www.mdpi.com/article/10.3390/life11121363/s1, Figure S1: Search terms used.

## References

[1] Hoffman J.I., Kaplan S., Liberthson R.R. (2004). Prevalence of congenital heart disease. Am. Heart J..

[2] Sairanen H.I., Nieminen H.P., Jokinen E.V. (2005). Late results and quality of life after pediatric cardiac surgery in Finland: A population-based study of 6,461 patients with follow-up extending up to 45 years. Semin. Thorac. Cardiovasc. Surgery Pediatr. Card. Surg. Annu..

[3] Jolley M., Colan S.D., Rhodes J., DiNardo J. (2015). Fontan Physiology Revisited. Anesthesia Analg..

[4] Gewillig M., Brown S.C. (2016). The Fontan circulation after 45 years: Update in physiology. Heart.

[5] Pekkan K., Aka I.B., Tutsak E., Ermek E., Balim H., Lazoglu I., Turkoz R. (2018). In vitro validation of a self-driving aortic-turbine venous-assist device for Fontan patients. J. Thorac. Cardiovasc. Surg..

[6] Backer C.L., Russell H.M., Pahl E., Mongé M.C., Gambetta K., Kindel S.J., Gossett J.G., Hardy C., Costello J.M., Deal B.J. (2013). Heart Transplantation for the Failing Fontan. Ann. Thorac. Surg..

[7] Schumacher K.R., Gossett J., Guleserian K., Naftel D.C., Pruitt E., Dodd D., Carboni M., Lamour J., Pophal S., Zamberlan M. (2015). Fontan-associated protein-losing enteropathy and heart transplant: A Pediatric Heart Transplant Study analysis. J. Heart Lung Transplant..

[8] Al Balushi A., Mackie A.S. (2019). Protein-Losing Enteropathy Following Fontan Palliation. Can. J. Cardiol..

[9] Simpson K.E., Cibulka N., Lee C.K., Huddleston C.H., Canter C.E. (2012). Failed Fontan heart transplant candidates with preserved vs impaired ventricular ejection: 2 distinct patient populations. J. Heart Lung Transplant..

[10] Khuong J.N., Wilson T.G., Grigg L.E., Bullock A., Celermajer D., Disney P., Wijesekera V.A., Hornung T., Zannino D., Iyengar A.J. (2020). Fontan-associated nephropathy: Predictors and outcomes. Int. J. Cardiol..

[11] Pundi K.N., Pundi K., Driscoll D.J., Dearani J.A., Li Z., Dahl S.H., Mora B.N., O’Leary P.W., Daly R.C., Cetta F. (2016). Heart transplantation after Fontan: Results from a surgical Fontan cohort. Pediatr. Transplant..

[12] Davies R.R., Sorabella R.A., Yang J., Mosca R.S., Chen J.M., Quaegebeur J.M. (2012). Outcomes after transplantation for “failed” Fontan: A single-institution experience. J. Thorac. Cardiovasc. Surg..

[13] Hernandez G.A., Lemor A., Clark D., Blumer V., Burstein D., Byrne R., Fowler R., Frischhertz B., Rn E.S., Schlendorf K. (2019). Heart transplantation and in-hospital outcomes in adult congenital heart disease patients with Fontan: A decade nationwide analysis from 2004 to 2014. J. Card. Surg..

[14] Jeon B.B., Park C.S., Yun T.-J. (2018). Heart Transplantation in Patients with Superior Vena Cava to Pulmonary Artery Anastomosis: A Single-Institution Experience. Korean J. Thorac. Cardiovasc. Surg..

[15] Michielon G., Van Melle J.P., Wolff D., Di Carlo D., Jacobs J.P., Mattila I.P., Berggren H., Lindberg H., Padalino M., Meyns B. (2015). Favourable mid-term outcome after heart transplantation for late Fontan failure. Eur. J. Cardio-Thorac. Surg..

[16] Tabarsi N., Guan M., Simmonds J., Toma M., Kiess M., Tsang V., Ruygrok P., Konstantinov I., Shi W., Grewal J. (2017). Meta-Analysis of the Effectiveness of Heart Transplantation in Patients with a Failing Fontan. Am. J. Cardiol..

[17] Page M.J., McKenzie J.E., Bossuyt P.M., Boutron I., Hoffmann T.C., Mulrow C.D., Shamseer L., Tetzlaff J.M., Akl E.A., Brennan S.E. (2021). The PRISMA 2020 statement: An updated guideline for reporting systematic reviews. BMJ.

[18] Doumouras B.S., Alba A., Foroutan F., Burchill L.J., Dipchand A.I., Ross H.J. (2016). Outcomes in adult congenital heart disease patients undergoing heart transplantation: A systematic review and meta-analysis. J. Heart Lung Transplant..

[19] Matsuda H., Ichikawa H., Ueno T., Sawa Y. (2017). Heart transplantation for adults with congenital heart disease: Current status and future prospects. Gen. Thorac. Cardiovasc. Surg..

[20] von Elm E., Altman D.G., Egger M., Pocock S.J., Gøtzsche P.C., Vandenbroucke J.P. (2007). The Strengthening the Reporting of Observational Studies in Epidemiology (STROBE) statement: Guidelines for reporting observational studies. PLoS Med..

[21] Assenza G.E., A Graham D., Landzberg M.J., Valente A.M., Singh M.N., Bashir A., Fernandes S., Mortele K.J., Ukomadu C., Volpe M. (2013). MELD-XI score and cardiac mortality or transplantation in patients after Fontan surgery. Heart.

[22] Berg C., Bauer B.S., Hageman A., Aboulhosn J.A., Reardon L.C. (2017). Mortality Risk Stratification in Fontan Patients Who Underwent Heart Transplantation. Am. J. Cardiol..

[23] Chrisant M., Naftel D., Drummond-Webb J., Chinnock R., Canter C., Boucek M., Boucek R., Hallowell S., Kirklin J., Morrow W. (2005). Fate of Infants with Hypoplastic Left Heart Syndrome Listed for Cardiac Transplantation: A Multicenter Study. J. Heart Lung Transplant..

[24] Hofferberth S.C., Singh T.P., Bastardi H., Blume E.D., Fynn-Thompson F. (2017). Liver abnormalities and post-transplant survival in pediatric Fontan patients. Pediatr. Transplant..

[25] Jayakumar K.A., Addonizio L.J., Kichuk-Chrisant M.R., Galantowicz M.E., Lamour J.M., Quaegebeur J.M., Hsu D. (2004). Cardiac transplantation after the Fontan or Glenn procedure. J. Am. Coll. Cardiol..

[26] Kaza A.K., Kaza E., Bullock E., Reyna S., Yetman A., Everitt M.D. (2015). Pulmonary vascular remodelling after heart transplantation in patients with cavopulmonary connection†. Eur. J. Cardio-Thoracic Surg..

[27] Marrone C., Ferrero P., Uricchio N., Sebastiani R., Vittori C., Ciuffreda M., Terzi A., Galletti L. (2017). The unnatural history of failing univentricular hearts: Outcomes up to 25 years after heart transplantation. Interact. Cardiovasc. Thorac. Surg..

[28] Mavroudis C., Deal B.J., Backer C.L., Stewart R.D., Franklin W.H., Tsao S., Ward K.M., DeFreitas R.A. (2007). 111 Fontan Conversions with Arrhythmia Surgery: Surgical Lessons and Outcomes. Ann. Thorac. Surg..

[29] Menachem J.N., Golbus J.R., Molina M., Mazurek J.A., Hornsby N., Atluri P., Fuller S., Birati E.Y., Kim Y.Y., Goldberg L.R. (2017). Successful cardiac transplantation outcomes in patients with adult congenital heart disease. Heart.

[30] Michielon G., Parisi F., Squitieri C., Carotti A., Gagliardi G., Pasquini L., Di Donato R.M. (2003). Orthotopic Heart Transplantation for Congenital Heart Disease: An Alternative for High-Risk Fontan Candidates?. Circualtion.

[31] Mital S., Addonizio L.J., Lamour J.M., Hsu D.T. (2003). Outcome of children with end-stage congenital heart disease waiting for cardiac transplantation. J. Heart Lung Transplant..

[32] Murtuza B., Dedieu N., Vazquez A., Fenton M., Burch M., Hsia T.-Y., Tsang V.T., Kostolny M. (2012). Results of orthotopic heart transplantation for failed palliation of hypoplastic left heart†. Eur. J. Cardio-Thorac. Surg..

[33] Murtuza B., Hermuzi A., Crossland D.S., Parry G., Lord S., Hudson M., Chaudhari M.P., Haynes S., O’Sullivan J.J., Hasan A. (2017). Impact of mode of failure and end-organ dysfunction on the survival of adult Fontan patients undergoing cardiac transplantation. Eur. J. Cardio-Thorac. Surg..

[34] Paniagua Martin M.J., Almenar L., Brossa V., Crespo-Leiro M.G., Segovia J., Palomo J., Delgado J., Gonzalez-Vilchez F., Manito N., Lage E. (2012). Transplantation for complex congenital heart disease in adults: A subanalysis of the Spanish Heart Transplant Registry. Clin Transplant..

[35] Petko M., Myung R.J., Wernovsky G., Cohen M.I., Rychik J., Nicolson S.C., Gaynor J.W., Spray T.L. (2003). Surgical reinterventions following the Fontan procedure. Eur. J. Cardio-Thorac. Surg..

[36] Rungan S., Finucane K., Gentles T., Gibbs H.C., Hu R., Ruygrok P.N. (2014). Heart transplantation in pediatric and congenital heart disease: A single-center experience. World J. Pediatr Congenit Heart Surg..

[37] Simpson K.E., Esmaeeli A., Khanna G., White F., Turnmelle Y., Eghtesady P., Boston U., Canter C.E. (2014). Liver cirrhosis in Fontan patients does not affect 1-year post-heart transplant mortality or markers of liver function. J. Heart Lung Transplant..

[38] Simpson K.E., Pruitt E., Kirklin J.K., Naftel D.C., Singh R., Edens R.E., Barnes A.P., Canter C.E. (2017). Fontan Patient Survival After Pediatric Heart Transplantation Has Improved in the Current Era. Ann. Thorac. Surg..

[39] Shi W.Y., Yong M., McGiffin D.C., Jain P., Ruygrok P.N., Marasco S.F., Finucane K., Keogh A., D’Udekem Y., Weintraub R.G. (2016). Heart transplantation in Fontan patients across Australia and New Zealand. Heart.

[40] van Melle J.P., Wolff D., Horer J., Belli E., Meyns B., Padalino M., Lindberg H., Jacobs J.P., Mattila I.P., Berggren H. (2016). Surgical options after Fontan failure. Heart.

[41] Griffiths E.R., Kaza A., von Ballmoos M.C.W., Loyola H., Valente A.M., Blume E.D., del Nido P. (2009). Evaluating Failing Fontans for Heart Transplantation: Predictors of Death. Ann. Thorac. Surg..

[42] Carrillo S.A., Texter K.M., Phelps C., Tan Y., McConnell P.I., Galantowicz M. (2021). Tricuspid Valve and Right Ventricular Function Throughout the Hybrid Palliation Strategy for Hypoplastic Left Heart Syndrome and Variants. World J. Pediatr. Congenit. Heart Surg..

[43] Marathe S.P., Zannino D., Cao J.Y., du Plessis K., Marathe S.S., Ayer J., Celermajer D.S., Gentles T.L., Sholler G.F., Justo R.N. (2020). Heterotaxy Is Not a Risk Factor for Adverse Long-Term Outcomes After Fontan Completion. Ann. Thorac. Surg..

[44] Schmiegelow M.D., Idorn L., Gislason G., Hlatky M., Køber L., Torp-Pedersen C., Sondergaard L. (2019). Cardiovascular complications in patients with total cavopulmonary connection: A nationwide cohort study. Int. J. Cardiol..

[45] Kovach J.R., Naftel D.C., Pearce F.B., Tresler M.A., Edens R.E., Shuhaiber J.H., Blume E.D., Fynn-Thompson F., Kirklin J.K., Zangwill S.D. (2012). Comparison of risk factors and outcomes for pediatric patients listed for heart transplantation after bidirectional Glenn and after Fontan: An analysis from the Pediatric Heart Transplant Study. J. Heart Lung Transplant..

[46] Alsoufi B., McCracken C., Kanter K., Shashidharan S., Border W., Kogon B. (2020). Outcomes of Multistage Palliation of Infants with Single Ventricle and Atrioventricular Septal Defect. World J. Pediatr. Congenit. Heart Surg..

[47] Cotter T.G., Wang J., Peeraphatdit T., Sandıkçı B., Ayoub F., Kim G., Te H., Jeevanandam V., Sabato D., Charlton M. (2021). Simultaneous Heart–Liver Transplantation for Congenital Heart Disease in the United States: Rapidly Increasing with Acceptable Outcomes. Hepatology.

[48] Daley M., Du Plessis K., Zannino D., Hornung T., Disney P., Cordina R., Grigg L., Radford D.J., Bullock A., D’Udekem Y. (2019). Reintervention and survival in 1428 patients in the Australian and New Zealand Fontan Registry. Heart.

[49] Kim M.H., Nguyen A., Lo M., Kumar S.R., Bucuvalas J., Glynn E.F., Hoffman M.A., Fischer R., Emamaullee J. (2021). Big Data in Transplantation Practice—the Devil Is in the Detail—Fontan-associated Liver Disease. Transplantation.

[50] Karikari Y., Abdulkarim M., Li Y., Loomba R.S., Zimmerman F., Husayni T. (2019). The Progress and Significance of QRS Duration by Electrocardiography in Hypoplastic Left Heart Syndrome. Pediatr. Cardiol..

[51] Moon J., Shen L., Likosky D.S., Sood V., Hobbs R.D., Sassalos P., Romano J.C., Ohye R.G., Bove E.L., Si M.-S. (2020). Relationship of Ventricular Morphology and Atrioventricular Valve Function to Long-Term Outcomes Following Fontan Procedures. J. Am. Coll. Cardiol..

[52] Stackhouse K.A., McCrindle B.W., Blackstone E.H., Rajeswaran J., Kirklin J.K., Bailey L.L., Jacobs M.L., Tchervenkov C.I., Jacobs J.P., Pettersson G.B. (2020). Surgical palliation or primary transplantation for aortic valve atresia. J. Thorac. Cardiovasc. Surg..

[53] Viegas M., Diaz-Castrillon C., Castro-Medina M., Silva L.D.F.D., Morell V.O. (2020). Bidirectional Glenn Procedure in Patients Less Than 3 Months of Age: A 14-Year Experience. Ann. Thorac. Surg..

[54] Serfas J.D., Thibault D., Andersen N.D., Chiswell K., Jacobs J.P., Jacobs M.L., Krasuski R.A., Lodge A.J., Turek J.W., Hill K.D. (2021). The Evolving Surgical Burden of Fontan Failure: An Analysis of the Society of Thoracic Surgeons Congenital Heart Surgery Database. Ann. Thorac. Surg..

[55] Schleiger A., Ovroutski S., Peters B., Schubert S., Photiadis J., Berger F., Kramer P. (2020). Treatment strategies for protein-losing enteropathy in Fontan-palliated patients. Cardiol. Young.

[56] Chaudhari M., Sturman J., O’Sullivan J., Smith J., Wrightson N., Parry G., Bolton D., Haynes S., Hamilton L., Hasan A. (2005). Rescue cardiac transplantation for early failure of the Fontan-type circulation in children. J. Thorac. Cardiovasc. Surg..

[57] Gamba A., Merlo M., Fiocchi R., Terzi A., Mammana C., Sebastiani R., Ferrazzi P. (2004). Heart transplantation in patients with previous Fontan operations. J. Thorac. Cardiovasc. Surg..

[58] Mitchell M.B., Campbell D.N., Ivy D., Boucek M.M., Sondheimer H.M., Pietra B., Das B.B., Coll J.R. (2004). Evidence of pulmonary vascular disease after heart transplantation for Fontan circulation failure. J. Thorac. Cardiovasc. Surg..

[59] Kanter K.R. (2016). Heart Transplantation in Children after a Fontan Procedure: Better than People Think. Semin. Thorac. Cardiovasc. Surgery: Pediatr. Card. Surg. Annu..

[60] Lin S.-N., Huang S.-C., Chen Y.-S., Chih N.-H., Wang C.-H., Chou N.-K., Yu H.-Y., Wu I.-H., Shun C.-T., Wang S.-S. (2016). Case Series: Heart Transplantation after Fontan Operation—Single-Center Experience. Transplant. Proc..

[61] Zhang Y., Alonso-Coello P., Guyatt G.H., Yepes-Nuñez J.J., Akl E.A., Hazlewood G., Pardo-Hernandez H., Etxeandia-Ikobaltzeta I., Qaseem A., Williams J.W. (2019). GRADE Guidelines: Assessing the certainty of evidence in the importance of outcomes or values and preferences—Risk of bias and indirectness. J. Clin. Epidemiol..

[62] (2020). Review Manager (RevMan).

[63] Stephens E.H., Tannous P., Mongé M.C., Eltayeb O., Devlin P.J., Backer C.L., Forbess J.M., Pahl E. (2020). Normalization of hemodynamics is delayed in patients with a single ventricle after pediatric heart transplantation. J. Thorac. Cardiovasc. Surg..

[64] Alsoufi B., Deshpande S., McCracken C., Kogon B., Vincent R., Mahle W., Kanter K. (2015). Results of heart transplantation following failed staged palliation of hypoplastic left heart syndrome and related single ventricle anomalies. Eur. J. Cardio-Thoracic Surg..

[65] Alsoufi B., Mahle W.T., Manlhiot C., Deshpande S., Kogon B., McCrindle B.W., Kanter K. (2016). Outcomes of heart transplantation in children with hypoplastic left heart syndrome previously palliated with the Norwood procedure. J. Thorac. Cardiovasc. Surg..

[66] Alsoufi B., Deshpande S., McCracken C., Kogon B., Vincent R., Mahle W., Kanter K. (2015). Outcomes and risk factors for heart transplantation in children with congenital heart disease. J. Thorac. Cardiovasc. Surg..

[67] Bando K., Turrentine M.W., Sun K., Sharp T.G., Caldwell R.L., Darragh R.K., Ensing G.J., Cordes T.M., Flaspohler T., Brown J.W. (1996). Surgical management of hypoplastic left heart syndrome. Ann. Thorac. Surg..

[68] Bernstein D., Naftel D., Chin C., Addonizio L., Gamberg P., Hsu D., Blume E., Canter C., Morrow R., Kirklin J. (2004). Outcome of listing for cardiac transplantation (Tx) for failed fontan: A follow-up multi-institutional study. J. Heart Lung Transplant..

[69] Carey J.A., Hamilton J.R., Hilton C.J., Dark J.H., Forty J., Parry G., Hasan A. (1998). Orthotopic cardiac transplantation for the failing Fontan circulation. Eur. J. Cardio-Thorac. Surg..

[70] D’Souza B.A., Fuller S., Gleason L.P., Hornsby N., Wald J., Krok K., Shaked A., Goldberg L.R., Pochettino A., Olthoff K.M. (2017). Single-center outcomes of combined heart and liver transplantation in the failing Fontan. Clin. Transplant..

[71] Eilers B., Albers E., Law Y., McMullan D.M., Shaw D., Kemna M. (2016). Posterior reversible encephalopathy syndrome after pediatric heart transplantation: Increased risk for children with preexisting Glenn/Fontan physiology. Pediatr. Transplant..

[72] Fullerton D.A., Campbell D.N., Jones S.D., Jaggers J., Brown J.M., Wollmering M.M., Grover F.L., Mashburn C., Luna M., Sondheimer H.M. (1995). Heart transplantation in children and young adults: Early and intermediate-term results. Ann. Thorac. Surg..

[73] Givertz M.M. (2015). Assessing the liver to predict outcomes in heart transplantation. J. Heart Lung Transplant..

[74] Iyengar A.J., Sharma V., Weintraub R.G., Shipp A., Brizard C.P., D’Udekem Y., Konstantinov I.E. (2014). Surgical strategies to facilitate heart transplantation in children after failed univentricular palliations: The role of advanced intraoperative surgical preparation. Eur. J. Cardio-Thorac. Surg..

[75] Izquierdo M., Almenar L., Martínez-Dolz L., Moro J., Agüero J., Sanchez-Lázaro I., Cano O., Ortiz V., Sánchez R., Salvador A. (2007). Mortality After Heart Transplantation in Adults with Congenital Heart Disease: A Single-Center Experience. Transplant. Proc..

[76] Jacobs J.P., Quintessenza J.A., Chai P.J., Lindberg H.L., Asante-Korang A., McCormack J., Dadlani G., Boucek R.J. (2006). Rescue cardiac transplantation for failing staged palliation in patients with hypoplastic left heart syndrome. Cardiol. Young.

[77] Kanter K.R., Mahle W.T., Vincent R.N., Berg A.M., Kogon B.E., Kirshbom P.M. (2011). Heart Transplantation in Children with a Fontan Procedure. Ann. Thorac. Surg..

[78] Kanter K.R., Vincent R.N., E Miller B., McFadden C. (1993). Heart transplantation in children who have undergone previous heart surgery: Is it safe?. J. Heart Lung Transplant..

[79] Lamour J.M., Kanter K.R., Naftel D.C., Chrisant M.R., Morrow W.R., Clemson B.S., Kirklin J.K. (2009). The Effect of Age, Diagnosis, and Previous Surgery in Children and Adults Undergoing Heart Transplantation for Congenital Heart Disease. J. Am. Coll. Cardiol..

[80] Lewis M., Ginns J., Schulze C., Lippel M., Chai P., Bacha E., Mancini D., Rosenbaum M., Farr M. (2016). Outcomes of Adult Patients With Congenital Heart Disease After Heart Transplantation: Impact of Disease Type, Previous Thoracic Surgeries, and Bystander Organ Dysfunction. J. Card. Fail..

[81] Menkis A.H., McKenzie F., Novick R.J., Kostuk W.J., Pflugfelder P.W., Goldbach M., Rosenberg H. (1991). Expanding applicability of transplantation after multiple prior palliative procedures. Ann. Thorac. Surg..

[82] Michielon G., Carotti A., Pongiglione G., Cogo P., Parisi F. (2011). Orthotopic Heart Transplantation in Patients with Univentricular Physiology. Curr. Cardiol. Rev..

[83] Michielon G., Parisi F., Di Carlo D., Squitieri C., Carotti A., Buratta M., Di Donato R.M. (2003). Orthotopic heart transplantation for failing single ventricle physiology. Eur. J. Cardio-Thorac. Surg..

[84] Miller J.R., Simpson K.E., Epstein D.J., Lancaster T.S., Henn M.C., Schuessler R.B., Balzer D.T., Shahanavaz S., Murphy J.J., Canter C.E. (2016). Improved survival after heart transplant for failed Fontan patients with preserved ventricular function. J. Heart Lung Transplant..

[85] Mitchell M.B., Campbell D.N., Boucek M.M. (2004). Heart transplantation for the failing Fontan circulation. Semin. Thorac. Cardiovasc. Surgery: Pediatr. Card. Surg. Annu..

[86] Mueller M.F., Paul A.C., Mann V., Koerner C.M., Valeske K., Thul J., Mazhari N., Bauer J., Schranz D., Akintuerk H. (2019). Anesthesia for Pediatric Heart Transplantation: Are Patients with a Failing Hemi-Fontan- or Fontan-Physiology Different?. Semin. Cardio-Thorac. Vasc. Anesth..

[87] Navaratnam M., Ng A., Williams G.D., Maeda K., Mendoza J.M., Concepcion W., Hollander S.A., Ramamoorthy C. (2016). Perioperative management of pediatric en-bloc combined heart-liver transplants: A case series review. Pediatr. Anesthesia.

[88] Pawlak S., Przybylski R., Skalski J., Śliwka J., Kansy A., Grzybowski A., Wierzyk A., Białkowski J., Maruszewski B., Zembala M. (2018). First Polish analysis of the treatment of advanced heart failure in children with the use of BerlinHeart EXCOR mechanical circulatory support. Kardiol. Polska.

[89] Pereira N.L., Shirali G. (2005). Cardiac Transplant Following Failed Fontan or Glenn Procedures. J. Am. Coll. Cardiol..

[90] Polyviou S., O’Sullivan J., Hasan A., Coats L. (2018). Mortality Risk Stratification in Small Patient Cohorts: The Post-Fontan Heart Transplantation Paradigm. Am. J. Cardiol..

[91] Razzouk A.J., Bailey L.L. (2014). Heart Transplantation in Children for End-Stage Congenital Heart Disease. Semin. Thorac. Cardiovasc. Surgery: Pediatr. Card. Surg. Annu..

[92] Reardon L.C., DePasquale E.C., Tarabay J., Cruz D., Laks H., Biniwale R.M., Busuttil R.W., Kaldas F.M., Saab S., Venick R.S. (2018). Heart and heart-liver transplantation in adults with failing Fontan physiology. Clin. Transplant..

[93] Schumacher K.R., Almond C., Singh T.P., Kirk R., Spicer R., Hoffman T.M., Hsu D., Naftel D.C., Pruitt E., Zamberlan M. (2015). Predicting graft loss by 1 year in pediatric heart transplantation candidates: An analysis of the Pediatric Heart Transplant Study database. Circulation.

[94] Schumacher K.R., Yu S., Butts R., Castleberry C., Chen S., Edens E., Godown J., Johnson J., Kemna M., Lin K. (2019). Fontan-associated protein-losing enteropathy and post-heart transplant outcomes: A multicenter study. J. Heart Lung Transplant..

[95] Schure A.Y., Kussman B.D. (2010). Pediatric heart transplantation: Demographics, outcomes, and anesthetic implications*. Pediatr. Anesthesia.

[96] Seddio F., Gorislavets N., Iacovoni A., Cugola D., Fontana A., Galletti L., Terzi A., Ferrazzi P. (2012). Is heart transplantation for complex congenital heart disease a good option? A 25-year single centre experience†. Eur. J. Cardio-Thorac. Surg..

[97] Serfas J.D., Patel P.A., Krasuski R.A. (2018). Heart Transplantation and Mechanical Circulatory Support in Adults with Congenital Heart Disease. Curr. Cardiol. Rep..

[98] Sglimbea A., Opriş M., Suciu M., Ispas M., Deac R., Suciu H. (2013). Indication for transplantation in a patient with univentricular heart. Chirurgia.

[99] Tjan T.D., Scheld H.H., Schmid C. (2006). Heart transplantation after Fontan’s procedure with bilateral cavopulmonary connections. Thorac. Cardiovasc. Surg..

[100] Vaikunth S.S., Concepcion W., Daugherty T., Fowler M., Lutchman G., Maeda K., Rosenthal D., Teuteberg J., Woo Y.J., Lui G.K. (2019). Short-term outcomes of en bloc combined heart and liver transplantation in the failing Fontan. Clin. Transplant..

[101] Voeller R.K., Epstein D.J., Guthrie T.J., Gandhi S., Canter C.E., Huddleston C.B. (2012). Trends in the Indications and Survival in Pediatric Heart Transplants: A 24-year Single-Center Experience in 307 Patients. Ann. Thorac. Surg..

[102] Kinsella A., Rao V., Fan C., Manlhiot C., Stehlik J., Ross H., Alba A.C. (2020). Post-transplant survival in adult congenital heart disease patients as compared to dilated and ischemic cardiomyopathy patients; an analysis of the thoracic ISHLT registry. Clin. Transplant..

[103] Rossano J.W., Singh T.P., Cherikh W.S., Chambers D.C., Harhay M.O., Hayes D., Jr Hsich E., Khush K.K., Meiser B., Potena L. (2019). The International Thoracic Organ Transplant Registry of the International Society for Heart and Lung Transplantation: Twenty-second pediatric heart transplantation report–2019; Focus theme: Donor and recipient size match. J. Heart Lung Transplant..

[104] Rodriguez de Santiago E., Tellez L., Garrido-Lestache Rodriguez-Monte E., Garrido-Gomez E., Aguilera-Castro L., Alvarez-Fuente M., Del Cerro M.J., Albillos A. (2020). Fontan protein-losing enteropathy is associated with advanced liver disease and a proinflammatory intestinal and systemic state. Liver Int..

[105] Grutter G., Di Carlo D., Gandolfo F., Adorisio R., Alfieri S., Michielon G., Carotti A., Pongiglione G. (2012). Plastic Bronchitis After Extracardiac Fontan Operation. Ann. Thorac. Surg..

[106] Fauziah M., Lilyasari O., Liastuti L.D., Rahmat B. (2018). Systemic ventricle morphology impact on ten-year survival after Fontan surgery. Asian Cardiovasc. Thorac. Ann..

[107] Julsrud P.R., Weigel T.J., A Van Son J., Edwards W.D., Mair D.D., Driscoll D.J., Danielson G.K., Puga F.J., Offord K.P. (2000). Influence of ventricular morphology on outcome after the Fontan procedure. Am. J. Cardiol..

[108] Pundi K.N., Pundi K., Johnson J.N., Dearani J.A., Li Z., Driscoll D.J., Wackel P.L., McLeod C.J., Cetta F., Cannon B.C. (2017). Sudden cardiac death and late arrhythmias after the Fontan operation. Congenit. Heart Dis..

[109] De Vadder K., Van De Bruaene A., Gewillig M., Meyns B., Troost E., Budts W. (2014). Predicting outcome after Fontan palliation: A single-centre experience, using simple clinical variables. Acta Cardiol..

[110] Rösner A., Khalapyan T., Dalen H., McElhinney D.B., Friedberg M.K., Lui G.K. (2018). Classic-Pattern Dyssynchrony in Adolescents and Adults with a Fontan Circulation. J. Am. Soc. Echocardiogr..

[111] De Groot N.M.S., Bogers A. (2017). Development of Tachyarrhythmias Late After the Fontan Procedure: The Role of Ablative Therapy. Card. Electrophysiol. Clin..

[112] Mizuno M., Ohuchi H., Matsuyama T.-A., Miyazaki A., Ishibashi-Ueda H., Yamada O. (2016). Diverse multi-organ histopathologic changes in a failed Fontan patient. Pediatr. Int..

[113] Hollander S.A., Cantor R.S., Sutherland S.M., Koehl D.A., Pruitt E., McDonald N., Kirklin J.K., Ravekes W.J., Ameduri R., Chrisant M. (2019). Renal injury and recovery in pediatric patients after ventricular assist device implantation and cardiac transplant. Pediatr. Transplant..

[114] Potena L., Zuckermann A., Barberini F., Aliabadi-Zuckermann A. (2018). Complications of Cardiac Transplantation. Curr. Cardiol. Rep..

[115] Di Nora C., Paldino A., Miani D., Finato N., Pizzolitto S., De Maglio G., Igor V., Sandro S., Chiara N., Gianfranco S. (2019). Transplantation in Kearns-Sayre Syndrome. Transplantation.

[116] Di Nora C., Livi U. (2020). Heart transplantation in cardiac storage diseases: Data on Fabry disease and cardiac amyloidosis. Curr. Opin. Organ. Transplant..

[117] de Diego-Sola A., Egues Dubuc C.A., Goena Vives C., Intxausti Irazabal J.J., Maiz Alonso O., Cobo Belaustegi M. (2021). Heart Transplantation in Systemic Sclerosis: A Therapeutic Option. Presentation of a Case and Literature Review. Reumatol. Clin. (Engl. Ed.).

