# Supplementary material for: Failures of the Fontan System in Univentricular Hearts and Mortality Risk in Heart Transplantation: A Systematic Review and Meta-Analysis
Source: Life (Basel). 2021 Dec 8;11(12):1363. doi: 10.3390/life11121363 (PMC8709145; doi:10.3390/life11121363)
Supplement: Supplementary file 1 [file life-11-01363-s001.zip › life-1426894-supplementary.pdf]

Supplementary Material

-(((Adolescent, Adult, Adult children, Child, Child, Preschool, Male, Female, Humans, Infant, Newborn, congenital[MeSH term) and ,("congenital heart disease, congenital heart diseases and pediatric", "congenital heart diseases and pediatric cardiology" [All Fields], and (Fontan procedure", "Fontan Procedure / methods", "Fontan procedure/ mortality", "Fontan procedure/therapy", "Fontan procedure/ therapy use" [MeSH Term]) OR ("Heart diseases", "Heart diseases/risk factors", "Heart diseases/ embryology", "Heart diseases/ congenital", "Heart diseases/physiopathology" , "Heart diseases/classification" , "Heart6 diseases/ mortality", "Heart diseases/ genetic" [MeSH Term]) OR ("Univentricular heart", "Univentricular heart/ mortality", "Univentricular heart/ pathology", "Univentricular heart/ physiology", "Univentricular heart/ surgery", "Univentricular heart/genetics"[MeSH]) OR ("Heart transplantation/ adverse effects", "Heart transplantation/ etiology", "Heart transplantation/ mortality", "Heart transplantation/ physiology", "Heart transplantation/ therapeutic use", "Heart transplantation/ therapy"[MeSH Term]) OR ("Heart valve diseases", "Heart valve diseases/ congenital", "Heart valve diseases/ classification", "Heart valve diseases/ complications", "Heart valve diseases/ genetics", "Heart valve diseases/ mortality", "Heart valve diseases/ Physiology", "Heart valve diseases/ physiopathology", "Heart valve diseases/ surgery", "Heart valve diseases/ transplantation"[MeSH Terms]), or ("Norwood procedure", "Norwood procedure/ mortality", "Norwood procedure/ adverse effects" [MeSH Term]) OR ("Heart defects, congenital/ anatomy and histology", "Heart defects, congenital/ classifications", "Heart defects, congenital/ complications", "Heart defects, congenital/ diagnostic", "Heart defects, congenital/ mortality", "Heart defects, congenital/pathology", "Heart defects, congenital/ physiology", "Heart defects, congenital/ physiopathology", "Heart defects, congenital/ surgery", "Heart defects, congenital/ therapy" [MeSH Terms]) AND ("Failing Fontan", "Failing Fontan circuit", "Failing Fontan circulations", "Failing Fontan operation", "Failing Fontan patient", "Failing Fontan physiology" [All Fields]) AND ("Abnormality heart", "Ventricles/abnormalities", "Ventricles/ surgery" [All Fields]) AND ("Congenitally Corrected Transposition Of The Great Arteries", "Aortico Left Ventricular Tunnel", "Double Inlet Left Ventricle" "Double Outlet Left Ventricle", "Hidouble Outlet Right Ventricle", "Hypoplastic Left Heart", "Right Ventricle", "Ventricular Septal Defect", "Pulmonary Atresia", "Mitral Atresia"[All Fields])
("arrhythmia, sinus", "arrhythmia, sinus/ anatomy, and histology", "arrhythmia, sinus/ classification", "Arrhythmia, sinus/ complications", "arrhythmia, sinus/ congenital" ,, "arrhythmia, sinus/ diagnosis", "arrhythmia, sinus/ etiology","arrhythmia, sinus mortality", "arrhythmia, sinus/ pathology", "arrhythmia, sinus/ physiology", "arrhythmia, sinus/ physiopathology", "arrhythmia, sinus/ surgery", "arrhythmia, sinus/ therapy" [MeSH Term]) arrythmia, cardiac", "arrythmia, cardiac/adverse effectsarrythmia, cardiac/ anatomy and histology, "arrythmia, cardiac/ classification", "arrythmia, cardiac/ complications", "arrythmia, cardiac/ congenital", "arrythmia, cardiac/ diagnosis", "arrythmia, cardiac/ etiology", "arrythmia, cardiac/ mortality", "arrythmia, cardiac/ pathology", "arrythmia, cardiac/ physiology", "arrythmia, cardiac/ physiopathology", "arrhythmia, cardiac/ surgery", "arrhythmia, cardiac/ therapy [MeSH Term]" OR ("Bronchitis / diagnosis", "Bronchitis / drug therapy", "Bronchitis / complications", "Bronchitis / congenital", "Bronchitis / mortality" [MeSH Term]) OR ("Heart failure", "Heart failure/ classification", "Heart failure/ complications", "Heart failure/ congenital" "Heart failure/ diagnosis", "Heart failure/ embryology", "Heart failure, diastolic/ surgery", "Heart failure, diastolic/ mortality", "Heart failure, systolic/ surgery", "Heart failure, systolic/ mortality" [MeSH Term]) OR ("Kidney", "acute kidney"),("Kidney Transplantation /

mortality”, “Kidney Transplantation / pathology”, “Kidney Transplantation / physiology” [MeSH Term]), AND (“Kidney Tubular Necrosis, Acute / epidemiology” [MeSH Term]) AND (“Kidney failure, chronic/ classification”, “Kidney failure, chronic/ complications”, “Kidney failure, chronic/ diagnosis”, “Kidney failure, chronic/ etiology”, “Kidney failure, chronic/ mortality”, “Kidney failure, chronic/ Physiology”, “Kidney failure, chronic/ Physiopathology”, “Kidney failure, chronic/ pathology”, “Kidney failure, chronic/ Surgery”, “Kidney failure, chronic/ Therapy” [MeSH Term]) OR (“Protein losing enteropathies”, “Protein losing enteropathies/ anatomy”, “Protein losing enteropathies/ classification”, “Protein losing enteropathies/ congenital”, “Protein losing enteropathies/ complications”, “Protein losing enteropathies/ diagnosis”, “Protein losing enteropathies/ etiology”, “Protein losing enteropathies/ metabolism”, “Protein losing enteropathies/ mortality”, “Protein losing enteropathies/ pathology”, “Protein losing enteropathies/ physiology”, “Protein losing enteropathies/ physiopathology”, “Protein losing enteropathies/ surgery”, “Protein losing enteropathies/ therapy” [MeSH Term])
“Death”,”, “Survival”, “Long Survival”, “Cardiac Survival”, “Risk Factors”, “Adverse Effects”, “Child Mortality”, “Child Mortalities”, “Alive”, “Cardiac Death”, “Sudden Cardiac Death”, “Infant Mortality”, “Adult Mortality”, “Cardiac Risk Factor”[All Fields]

Figure S1. Search terms used.
